# Linguistic disparities in cross-language automatic speech recognition transfer from Arabic to Tashlhiyt

**DOI:** 10.1038/s41598-023-50516-3

**Published:** 2024-01-03

**Authors:** Georgia Zellou, Mohamed Lahrouchi

**Affiliations:** 1 University of California, Davis, Davis, USA; 2 CNRS & Université Paris 8, Paris, France

**Keywords:** Psychology, Human behaviour

## Abstract

Tashlhiyt is a low-resource language with respect to acoustic databases, language corpora, and speech technology tools, such as Automatic Speech Recognition (ASR) systems. This study investigates whether a method of cross-language re-use of ASR is viable for Tashlhiyt from an existing commercially-available system built for Arabic. The source and target language in this case have similar phonological inventories, but Tashlhiyt permits typologically rare phonological patterns, including vowelless words, while Arabic does not. We find systematic disparities in ASR transfer performance (measured as word error rate (WER) and Levenshtein distance) for Tashlhiyt across word forms and speaking style variation. Overall, performance was worse for casual speaking modes across the board. In clear speech, performance was lower for vowelless than for voweled words. These results highlight systematic speaking mode- and phonotactic-disparities in cross-language ASR transfer. They also indicate that linguistically-informed approaches to ASR re-use can provide more effective ways to adapt existing speech technology tools for low resource languages, especially when they contain typologically rare structures. The study also speaks to issues of linguistic disparities in ASR and speech technology more broadly. It can also contribute to understanding the extent to which machines are similar to, or different from, humans in mapping the acoustic signal to discrete linguistic representations.

## Introduction

The huge rise and fast-paced advancement of speech technology—computational systems that understand and generate spoken language—allows for millions of people to communicate with devices using speech to perform a range of tasks (i.e., dictate text messages, seek information using voice searches, play games or music, etc.)^1,2^. Speech-enabled devices can also be used for a wide range of education and healthcare applications, such as language translation^3^, language learning^4,5^, and “emergency media” technologies that connect users to emergency service providers in the case of a crisis^6^.

However, there are asymmetries in who has access to speech technology. Currently, there are over 7,000 languages spoken in the world^7^, but speech technology is available in only approximately 100 languages^8^. There is a bias in speech technology development for languages that have large digital resources, such as acoustic databases, lexicons and pronunciation dictionaries, and transcribed corpora^9^. Low-resource languages, like Tashlhiyt (an Amazigh language of Southern Morocco), are disadvantaged for speech technologies, such as Automatic Speech Recognition (ASR), which provide users with a range of applications for healthcare, education, and other domains where ASR is valuable. Even for languages that have commercially available ASR systems, there are disparities in how well they perform across dialects and varieties^10–12^. Access to ASR for more languages and varieties is beneficial to language communities all around the world by providing more equitable access to technology.

One approach to addressing the gaps in availability of speech technology is to re-use an existing acoustic model and ASR system developed for a high-resource language (the “source” language) that is phonologically similar to the “target” low-resource language without any kind of adaptation^13^. Prasad et al. argue that if two languages are similar enough in terms of their phonological inventories, the acoustic model of the source language could be applied to the low-resource target language without any modifications. They demonstrate this approach using Hindi as the source language and Marathi and Gujarati as the target languages and report a less than 20% word error rate (WER). Researchers have applied this technique for re-using ASR systems for other target and source languages (e.g.,^14^ for Tigrinya as target language using Amharic as source language;^15^ and^16^ use a variety of source and target languages, including Bulgarian and Czech with varying success). In the current study, we apply the cross-language ASR transfer method to Tashlhiyt, which is under-resourced with respect to speech technology, using Arabic as the source language.

In addition to this goal of determining whether cross-language re-use of ASR for Tashlhiyt is a viable solution, we are interested in exploring the linguistic, phonological, and phonetic factors that might influence the success of this approach. While much of the work in this area is aimed at understanding the broad practical challenges with this approach, examining systematic disparities in recognition of different phonological and phonetic forms of words within the target language can lead to more theoretically-guided and effective cross-language ASR transfer practices. In particular, the current study focuses on two types of systematic ways in which speech patterns vary across and within languages: phonotactic variation (the allowance of certain sound sequences in the words of a language) and speaking style variation (talking clearly vs. talking casually). Human listeners with different native language backgrounds show difficulty adapting to phonotactic patterns that are not allowed in their first language^17–19^. Do ASR systems also show “native” (i.e., source) language phonotactic biases when adapting to a target language? This is a gap in our understanding of the parallels and differences across machine and human cross-language perception and can identify major disparities that could arise during transfer of any kind.

In this study, we specifically focus on Tashlhiyt, which is well-known in the linguistics literature for having typologically rare phonological patterns. Tashlhiyt permits many words that contain sequences of only consonants—*vowelless* words (e.g., *zdm* ‘to collect wood’; See Supplementary Materials for our wordlist)^20–24^. A fundamental observation is that certain sequences of speech sounds are favored, and others dispreferred, in the forms of words found cross-linguistically. Across languages of the world, there is an overwhelming preference for words to contain a vowel. One view is that vowelless words are dispreferred because they might be harder for listeners to hear, since a lack of vowels means less robust acoustic cues in the speech signal. Thus, it has been argued that observed cross-linguistic phonological tendencies are the result of auditory properties of the speech signal or perceptual processing mechanisms^25,26^. Do the same biases apply to an ASR system? It might be predicted that since ASR systems are trained to detect variations in spectral amplitude when identifying the signal^27^, vowelless words will be more difficult for speech recognition systems since they contain the smallest differences in acoustic modulations across segments, compared to words that have vowels which contain large acoustic perturbations^28,29^. Understanding the role of word form variation on ASR recognition is a major area of work with both scientific and commercial application^27^. Since Tashlhiyt has unusual phonological patterns, the current study can enhance our understanding of how ASR handles even the rarest cross-linguistic variation.

### Target and source languages: Tashlhiyt and Arabic

There is currently no commercially available speech recognition technology system available for Tashlhiyt. Some researchers have worked on developing speech recognition systems for other Amazigh languages. For instance^30^, used open-source technology (CMU-Sphinx) to create an ASR system for Tarifit, a language related to Tashlhiyt. However, Telmem and Ghanou built their system on limited acoustic data from 1 speaker, and report that their system is highly speaker-dependent. A speaker-independent speech recognition system for spoken digits and letters in Amazigh languages has also been developed^31^. These systems are limited in their generalizability across takers and words and also are not easily accessible by most people for every-day use. Moreover, neither of these studies discuss the systems’ performance for Tashlhiyt, or on vowelless words, specifically.

The cross-language ASR transfer approach^13^ has high potential to create a successful ASR system for Tashlhiyt. In the current study, we use a language model built for Arabic using Sonix Speech-to-text, a popular and versatile online transcription service. Arabic and Tashlhiyt both have three vowels and a large consonant inventory, with overlapping phoneme inventories: i.e., pharyngealized consonants, uvular, and pharyngeal segments; yet, there are major differences in the phonotactics, or allowable sound sequences, between Tashlhiyt and Arabic, such as the presence of vowelless words in Tashlhiyt which is not permitted in Arabic^20,24,32,33^.

ASR systems convert speech to text transcriptions. Amazigh languages have their own ancient writing system (Tifinagh), which was selected as the official orthography for those languages in Morocco in 2003, though Arabic was and is still often used for writing Amazigh^34^. Using the Arabic ASR system means converting Tashlhiyt speech into Arabic orthographic forms, which will be familiar to many Amazigh speakers (though, we acknowledge, comes with complex social, political, and cultural concerns). A practical benefit of using Arabic speech to text is that there is a large amount of shared vocabulary and cognates between Tashlhiyt and Arabic^34^, which can be useful given our non-modification ASR transfer approach in the current study. Arabic is an alphabet with characters representing consonants and long vowels. Optionally, diacritics can be added to characters to indicate short vowels and geminates; the output of the ASR system used in the current study does not use diacritics in the transcriptions. Thus, the use of Arabic transcriptions is appropriate since we are interested in the present study on recognition of words without vowels.

By applying the cross-language ASR transfer approach to Tashlhiyt, we can investigate fundamental questions about how variation in word forms across source and target language impacts the efficacy of cross-language ASR adaptation. Examining whether a current acoustic model can be re-used for Tashlhiyt, and for vowelless words in Tashlhiyt in particular, can be a key milestone in the development of ASR technology for the low-resource language. Moreover, identification of the specific types of phonological structures, speaking styles, and phonetic patterns that might pose particular challenges using this approach can also be used to support targeted adaptation and fine-tuning when re-using an existing acoustic model to a new language in other source and target language contexts.

### ASR performance across speaking styles

As mentioned above, we also explore the effect of speech style variation on the efficacy of this cross-language ASR transfer approach. In particular, we compare ASR transcription errors across clear and casual (reduced) speech productions of Tashlhiyt words. Speakers vary their speaking style based on communicative context: they can hyperarticulate and produce more extreme acoustic realizations of words if they believe the listener will have trouble understanding or they can exert less articulatory effort and produce more acoustically reduced forms of words if they think the listener will have no trouble comprehending^35–37^. Clear speech forms are indeed more intelligible and better perceived by listeners^38,39^. ASR systems, also, have been shown to perform better at word recognition of clear speech forms, compared to reduced forms^40,41^. Temporal and segmental reduction are commonly observed pronunciation variants in reduced speech that lead to ASR errors across languages^42,43^.

How can this speaking style disparity be addressed? Increasing the amount of casual speech the ASR systems get in training can improve recognition accuracy for reduced word forms^42,44^. Another solution is to add sequential pronunciation variants to the pronunciation dictionary, e.g. a “clear” and a “casual” form of variable words^42,43^. But first, understanding the nature of disparities across speech styles in ASR performance when adapting to a low-resource language can be helpful in targeting the type of data that could be used to efficiently improve the model. Clear speech does not always contain the same types of acoustic enhancements across languages and varieties^37,45^, therefore, it is critical to explore the performance of different types of registers when exploring the efficacy of cross-language ASR transfer.

### Current study

The current study tests performance of the “out-of-the-box” cross-language ASR transfer method on Tashlhiyt vowelless and voweled words, produced in both Clear and Casual speaking styles. This study was designed to address three major gaps in the literature on speech recognition technology.

First, this study addresses a gap in understanding how cross-linguistic differences in phonological structure influence ASR transfer. If there are disparities across voweled and vowelless words for Tashlhiyt, this can illuminate how *phonotactic* differences (differences in word shape and sound combinations) across target and source languages can result in less effective ASR transfer even if the languages have similar phoneme inventories. This is also a gap in our understanding of the parallels or differences across machine and human cross-language perception. Human adults show particular difficulties in learning second languages that differ from their native language’s restrictions on sound sequences^18^. Therefore, we predict ASR systems will have a hard time identifying words in the target language that do not conform to the source language’s rules of sound sequencing and word structure.

Second, there is a gap in examining how speaking style differences influence cross-language ASR transfer. Thus, we also compare cross-language ASR transfer across clear and reduced speech productions of the target language. It is predicted that the ASR system will perform less well for reduced speech forms, consistent with prior findings that fast, casual speech is less well understood than clear speech by human (e.g., for Tashlhiyt^46^) and machine^42^ comprehenders. However, if vowelless words are understood at an even lower rate than voweled words in one speech mode, this can further reveal how disparities across phonotactic patterns in the target language can be amplified given the range of variation in speaking styles that is found across users and contexts.

Finally, Tashlhiyt is one of the thousands of languages that do not have commercially-available speech technology systems. Exploring if cross-language ASR transfer is a viable approach to speech recognition for Tashlhiyt is one small step in addressing the huge gap in speech-enabled technology availability for under-resourced languages.

## Methods

### Target words

Target items were 74 Tashlhiyt words consisting of 37 vowelless words and 37 words with a vowel nucleus. The voweled words were selected to contain a vowel with consonants on either side. Within voweled words, there was roughly an equal number of items containing /a/ (n = 11), /i/ (n = 12), and /u/ (n = 14), the three vowel phonemes in Tashlhiyt.

The vowelless words were selected to contain exactly three consonants. We focus on tri-segmental vowelless words as a way to strategically home in on the precise mechanisms at play for ASR comprehension disparities across voweled and vowelless sequences: a trisegmental word form is the smallest structure where a middle segment is surrounded by two consonants. One way to quantify how much acoustic modulation a segment carries is sonority (defined as the relative loudness and resonant properties of a sound). All sounds can be assigned a ranking within a universal hierarchy of sonority: vowels, which are most sonorous, are assigned the highest numerical sonority score, and consonants are assigned sequentially lower values based on their acoustic-sonority properties ([47]8 = vowels, 7 = glides, 6 = liquids, 5 = nasals, 4 = voiced fricatives, 3 = voiceless fricatives; 2 = voiced stops; 1 = voiceless stops). As mentioned in the Introduction, vowelless words might be more difficult for speech recognition systems; since vowelless words contain smaller differences in acoustic modulations across segments,  it will be harder to detect variations in spectral amplitude in the acoustic signal^27^. Following from this, we also predict that *within* vowelless words, ASR performance will decrease for items that contain center segments with lower sonority values than those that contain center consonants with higher sonority values. We selected target vowelless word items that contained a range of sonority values of the center consonant (center segment sonority ranged from 6–1; glides are not permitted as word centers in Tashlhiyt).

A full list of the target words is provided in the Supplemental Materials.

### Stimulus materials

Four native Tashlhiyt speakers produced the wordlist in two speaking styles. Three of the speakers were born in Agadir, Morocco and one speaker was born outside of Marrakech, Morocco (mean age = 48 years old; 1 female, 3 male).

Recordings were made with Audacity. The recording took place in a sound attenuated booth using a microphone and audio mixer (AT 8010 Audio-technica microphone and USB audio mixer, M-Audio Fast Track), digitized at a 44.1 kHz sampling rate.

Words were recorded in two speaking styles: Clear and Casual. To elicit Clear Speech, the speakers were given instructions used to elicit clear speech in prior work^48^: “In this condition, speak the words clearly to someone who is having a hard time understanding you.” Following the Clear Speech style elicitation, the speakers produced the words in a fast, casual speaking style with the following instructions also modeled after those used in prior work^48^: “now, speak the list as if you are talking to a friend or family member you have known for a long time who has no trouble understanding you, and speak quickly”. The speakers produced the words in two frame sentences in each speaking style: *ini ___ jat tklit* ‘say ___ once’, *inna ___ baɦra* ‘he said ___ a lot’. A total of 1,185 target word productions were collected.

The study was approved by the UC Davis Institutional Review Board (IRB). All research was performed in accordance with guidelines and regulations of the IRB. Informed consent was obtained from the speakers.

### Data and coding

Each recording was transcribed using Sonix, an ASR tool for transcribing audio, with the language set to Arabic. The Sonix transcript for each file was downloaded as a .csv file, with a timestamp for each word. Transcriptions for target words were identified and coded based on the timestamp. Any punctuations were removed from the transcript.

Each target word was given an acceptable “ground truth” Arabic transcription (non-diacritized, as is the output of the ASR system); these written forms are provided in the Supplementary Materials. There are several consonantal phonemes (in both source and target languages) for which there is not a distinct Arabic letter. First, based on tests of the ASR system and conventions for how this letter is used across Arabic varieties, we used the Arabic letter *jeem* to represent the phoneme /g/ (e.g., “رجل” , < r-ž*-l > for /rgl/), in addition to /ž/ (e.g., “جلد”, < ž-l-d > for /žld/). Also, Tashlhiyt has additional pharyngealized segments that do not have a distinct letter in written Arabic. For these segments, the “ground truth” transcription contained the non-pharyngealized letter (e.g., “زور” < z-w-r > for /zˤurˤ/).

ASR transcription performance was assessed in three ways:

- **Transcription generated**: In a small number of cases, the ASR system did not generate a transcription of the target word, indicating a recognition failure—for instance, that the system treated the speech sample as noise or non-linguistic audio. Since vowelless words, in particular, might contain no voicing information, they might be particularly prone to being treated like “noise” rather than “speech” by the ASR system and hence not transcribed. Thus, we evaluated this as one metric of ASR performance—generating a transcription is evidence that the ASR treats the input as speech. All trials were coded as “transcription” or “no transcription”.
- **Transcription accuracy**: Of the words where a transcription was generated, these were then coded for accuracy if the ASR transcription output matched the “ground truth” transcription of the word in Arabic script or not.
- **Levenshtein distance**: For each transcribed word, Levenshtein distance was calculated, which quantifies the distance between two strings in terms of substitutions, deletions, and insertions^49^ (and used in linguistic studies such as for measures of phonetic distances between languages and varieties^50,51^). Levenshtein distance is used here as a metric for phonetic distance between ground truth and generated transcriptions.

We also coded ASR transcriptions as being either real Arabic words (identified as such using google translate) or nonwords. 83% of target word transcriptions generated by the ASR were real words. A chi-square test revealed that there was a difference in the proportion of real vs. nonwords for vowelless and voweled words (Χ^2^(1) = 82.5, *p* < 0.001). Vowelless words were less likely to be transcribed as nonwords (36/547 transcriptions) than voweled words (156/573 transcriptions).

## Results

### Comparing ASR performance across voweled and vowelless words

Our first set of analyses investigated whether the ASR performs differently for vowelless and voweled words, as well as the effect of speaking style.

We ran two separate mixed effects logistic regressions. The first model analyzed whether there was a difference in rates of “no transcription” across target words as a function of word type and speech style. For this model, data were coded for whether the ASR system produced any transcription (= 1) or not (= 0). The second model was run on a subset of data for which the ASR system did produce a transcription (n = 1,120). For these data, we modeled accuracy in generating the ground truth transcription (= 1) or not (= 0). Both models were run using the *glmer* function in the *lme4* R package^52^. The models included fixed effects of Word Type (voweled vs. vowelless) and Speaking Style (Clear vs. Casual), as well as their interaction. Effects were sum-coded. For the random effect structure: We first fit models with maximal random effects structure, consisting of random intercepts for speaker and word, as well as by-speaker random slopes for Word Type and Style and the interaction between them. If this resulted in a singularity error (indicating overfitting of the random effects), then the random effects structure was then simplified by removing predictors that accounted for the least amount of variance until the model fit^53^. (Random effects simplification for all models reported in this paper followed the same procedure.) The glmer syntax for the retained transcription presence model was: transcription presence ~ Word Type * Speech Style + (1 | Word) + (1 + Speech Style | Speaker); the glmer syntax for the retained accuracy model was: accuracy ~ Word Type * Speech Style + (1 | Word) + (1 + Word Type + Speech Style | Speaker).

**Figure 1:**
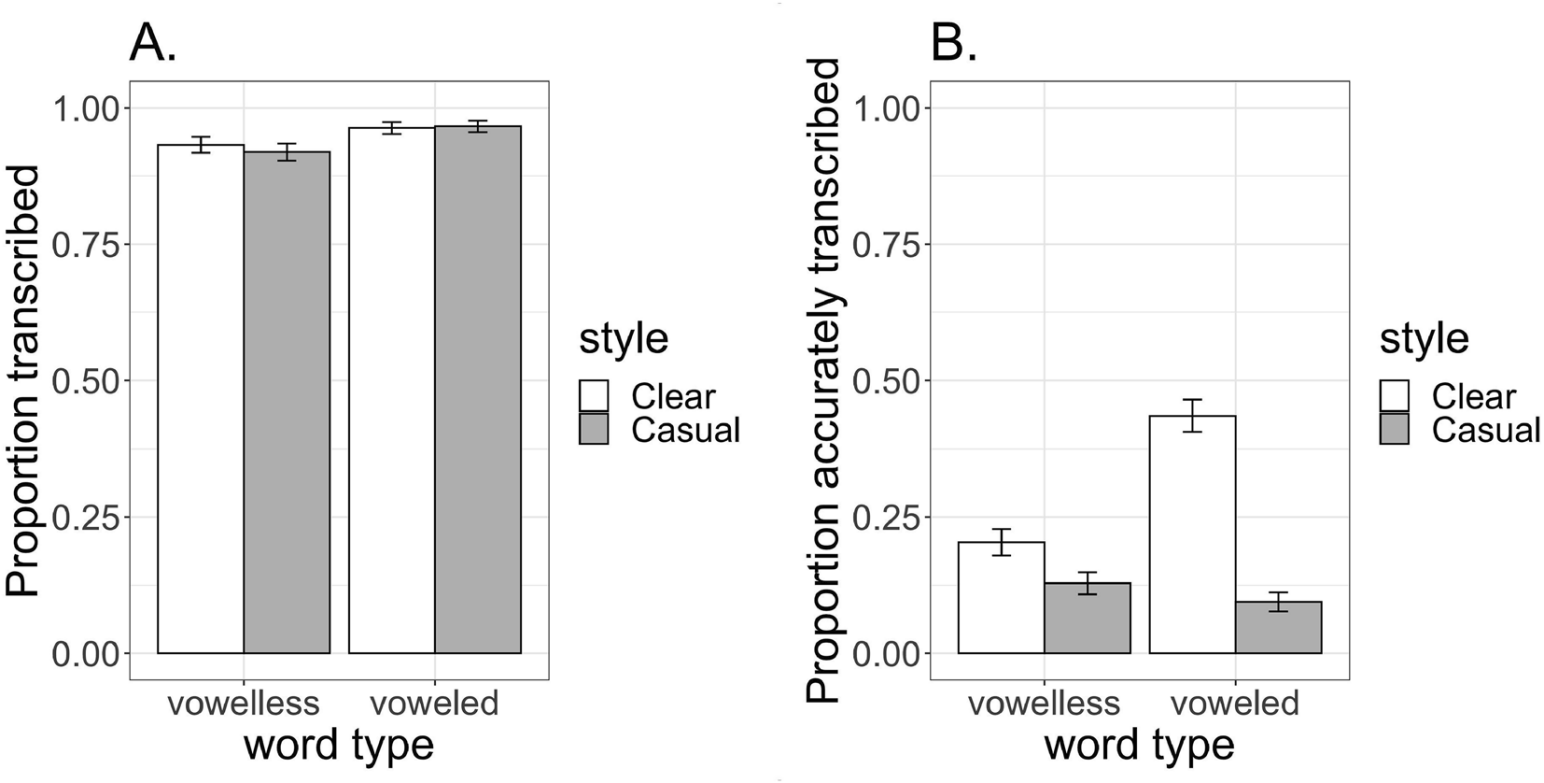
Mean performance (and standard errors) for generating a transcription (**A**) and (**B**) accuracy in reflecting the written Arabic “ground truth” for words where a transcription was generated.

Figure 1A shows the averaged performance of the ASR system in generating a transcription across vowelless and voweled words by speaking style. Overall, only 5% (n = 65) of target words did not get assigned a transcription by the ASR system. Yet, the transcription presence model revealed an effect of Word Type such that vowelless words were more likely to not get transcribed at all than voweled words (*coef.* = -0.4, SE = -0.2, *z* = -2.5, *p* < 0.05). Thus, even though 95% of target words did get transcribed, there were still disparities in this measure by word type. There was not an effect of Speaking Style (*p* = 0.7) and there was not an interaction between Word Type and Speaking Style (*p* = 0.5) for the transcription presence model.

Figure 1B presents accuracy data for items where a transcription was generated. Speaking Style predicted accuracy, with the ASR system more likely to generate a “ground truth” transcription of Clear forms of words (*coef*. = 0.8, SE = 0.1, *z* = 5.9, *p* < 0.001). There was also an interaction between Word Type and Speech Style (*coef*. = -0.5, SE = 0.1, *z* = -5.2, *p* < 0.001). To explore this interaction, a *Tukey’s* HSD pairwise comparison was performed using the *emmeans()* function in the emmeans R package^54^. This post hoc comparison revealed that while vowelless words were less accurately transcribed than voweled words in Clear speech (*coef*. = -1.5, SE = 0.4, *z* = -3.5, *p* < 0.001), there was no difference in transcription accuracy for word types in Casual speech (*p* = 0.4).

Next, we analyzed Levenshtein distances for the items where a transcription was generated using a mixed effects linear regression model. The model included fixed effects of Word Type (voweled vs. vowelless) and Speaking Style (Clear vs. Casual), as well as their interaction. Effects were sum-coded. The random effects structure of the model started as maximal, but was reduced to avoid overfitting (lmer syntax of retained model: distance ~ Word Type * Style + (1| Word) + (1|Speaker)).

**Figure 2:**
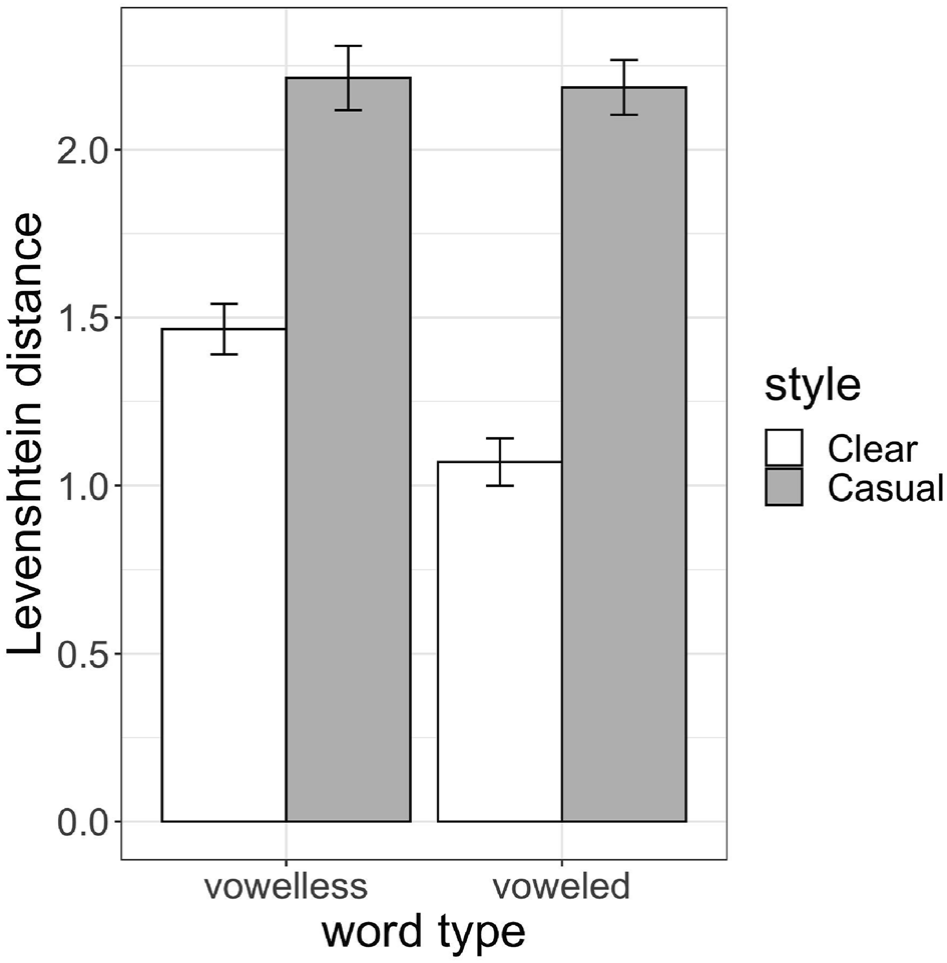
Levenshtein distance means (and standard errors) for transcribed words, averaged by Word Type and Speaking Style.

Figure 2 provides mean Levenshtein distances across items and conditions. The model computed an effect of Speaking Style wherein Clear speech transcriptions had lower Levenshtein distances than Casual speech productions (*coef.* = -0.5, SE = 0.04, *t* = -12.6, *p* < 0.001), meaning that transcriptions for casual items were further away from ground truth transcriptions than those for clear speech productions. There was also an interaction between Word Type and Style (*coef.* = 0.1, SE = 0.04, *t* = 2.7, *p* < 0.01). A post hoc pairwise comparison with emmeans revealed that Levenshtein distances were higher for vowelless words than for voweled words in Clear speech (*coef*. = 0.4, SE = 0.2, *t* = 2.6, *p* < 0.05), but there was no difference between word types in Casual speech (*p* = 0.9). In other words, within Clear speech, transcriptions for vowelless words were more different from ground truth forms than those for voweled words.

### ASR performance within vowelless words

Finally, we ran two analyses on a subset of the data from vowelless words only. As mentioned above, our vowelless items varied in having consonantal centers that are more sonorous (i.e., /l/ and /r/ are loud, resonant, and highly sonorant consonants) or less sonorous (e.g., /d/ and /k/ are less sonorous sounds). Using a numerical scale that assigns consonants a rating based on their sonority properties^47^, we tested whether the sonority value of the central segment in vowelless words predicts ASR transcription accuracy and Levenshtein distances.

First, we ran a mixed effects logistic regression model on accuracy values for vowelless words only with fixed effects of Sonority value of the center consonant (continuous variable from 1–6, centered) and Speech Style (Clear vs. Casual, sum-coded), and their interaction (glmer syntax for the retained model: accuracy ~ Speech Style * Center Consonant Sonority + (1 | Word) + (1 | Speaker)). Next, we ran a mixed effects linear regression on Levenshtein distance values for vowelless words with fixed effects of Sonority value, Speech Style, and their interaction (lmer syntax: distance ~ Style * Center Consonant Sonority + (1 | Word) + (1 + Speech Style | Speaker)).

For the accuracy model, there was an effect of Style, wherein Clear speech was more accurately transcribed than Casual speech (*coef*. = 0.3, SE = 0.1, *z* = 2.2, *p* < 0.05). There was also an interaction between Sonority and Style (*coef*. = 0.3, SE = 0.1, *z* = 2.7, *p* < 0.01), indicating that vowelless words containing more sonorous center consonants are more likely to be assigned the correct transcription by the ASR system in Clear speech; it also means the reverse is true: lower ASR accuracy for vowelless words with lower sonority centers.

For the Levenshtein model, the effect of sonority was associated with a negative coefficient (*coef.* = -0.3, SE = 0.1, *t* = -2.7, *p* < 0.05), meaning that vowelless words with less sonorant centers are more likely to be transcribed with forms that are further away from the ground truth transcriptions. There was not an interaction between Style and Sonority (*p* = 0.4).

Thus, these analyses provide converging evidence that, even within vowelless words, ASR performance decreases for Tashlhiyt words that contain less sonorant word centers.

## General discussion

There is a language gap in speech technology: computer systems that understand speech are only available in a fraction of the extant languages of the world. In the current study, we re-used an existing, commercially-available ASR system from Arabic without any modification for Tashlhiyt, a low-resource language with over seven million speakers. The segmental inventories of Arabic and Tashlhiyt are similar and using Arabic orthography allows for a great deal of flexibility in providing functional transcriptions of vowelless words in Tashlhiyt (even though Amazigh languages have their own writing system). Overall, our results indicate this is a promising approach to adapt an existing ASR system for an under-resourced language. Yet, we found disparities in ASR performance across items and speech styles in Tashlhiyt. There were three key findings that reflect systematic phonological and speaking style factors that affect the efficacy of this method.

First, even though the system did not generate a transcription for only 5% of tokens, the proportion of items where the ASR system failed to transcribe the target word was higher for vowelless words than for words that contained vowels. This indicates that the ASR system is more likely to ignore vowelless words than voweled words. One possible explanation is that vowelless words are more likely to be identified as non-speech than voweled words. ASR systems detect variations in spectral amplitude when identifying the signal^27^. So, vowelless words, which overall contain smaller differences in spectral modulations across segments, compared with large acoustic perturbations that occur from consonant to vowel, will indeed have a unique acoustic signature that might be interpreted as non-speech by a ‘naive’ ASR system and not transcribed.

Second, when the ASR generated a transcription, we found that the WER and Levenshtein distances were higher for casual, compared to clear, speaking styles. This is an unsurprising finding as ASR systems perform less well on reduced speech, even for languages that they are trained on and built for^55^, but here it highlights even further disparities that can arise using the cross-language ASR transfer method due to speaking style variation. While many practical issues arise when considering similarities between source and target language^13^, this is the first study, to our knowledge, investigating the effect of speaking style on cross-language ASR transfer.

Thirdly, in Clear speech, both WERs and Levenshtein distances between generated and ground truth transcriptions were greater for vowelless words than words with vowels. Note that there was no difference for these word forms in casual speech because performance was low across the board. Only in Clear speech, where ASR performance was enhanced, do we observe that vowelless words are accurately transcribed at lower rates than voweled words. This interaction parallels recent findings examining perceptual patterns of clearly- and casually-produced Tashlhiyt words by non-native listeners in^46^. In that study, Tashlhiyt-naive listeners showed a clear speech boost in perceiving Tashlhiyt words that have phonotactic patterns that are also present in English (i.e., clear speech enhanced the discrimination of pairs like *sin* vs. *fin*). But there was not a perceptual benefit for clear speech productions of word forms that are not present in English (e.g., clear speech provided no improvement for discrimination of word pairs like *ssin* vs. *fsin*). Thus, our observation that clear speech does not enhance the intelligibility of words that are illegal in the “native” language than for those that are legal has support for both human perception and machine speech recognition.

Our findings highlight linguistic disparities that may occur in cross-language ASR transfer: phonotactically rare word forms. Speech recognition systems not trained on vowelless words will fail: a vowelless word is more likely to be transcribed incorrectly, and it is more likely to be further from the ground truth form making it even harder for a user to understand. An explanation for this disparity can be attributed to differences in phonotactics across source and target language: Arabic does not permit vowelless words. This observation indicates that re-using an acoustic model trained on a source language with different phonotactic patterns can lead to disparities for phonological structures in the target language that are not attested in the source language. This means we can predict other cases where ASR transfer is likely to be problematic: even if target and source language have overlapping phoneme inventories, differences in the shapes of word forms across them might lead to disparities.

Furthermore, we also found that within vowelless words, ASR performance was lower for items containing center consonants that are less sonorous than words containing more sonorous centers. Thus, our results allow us to home in on which types of vowelless word forms, specifically, the ASR systems have the most trouble with: words with less resonant and sonorant center consonants. This observation provides even further evidence that ASR disparities are systematic and shows how machine recognition patterns can be analogous to auditory properties of human sound systems.

This study had several limitations that can serve as directions for future work. One limitation of the current study is that the speech samples were not elicited as device-directed speech. Prior work has observed that speakers make distinct clear speech adjustments when talking to ASR-enabled devices, like smartphones and voice-AI assistants^56,57^, and adjust their pronunciations even more when the machine makes an error^39^. A ripe future direction is to explore whether cross-language ASR re-use recognition accuracy improves if the speakers are producing authentic device-directed speech. There are other factors that can be explored in future studies, such as how ASR transfer performs when there is background noise or other types of environmental factors (e.g., multiple talkers). Particularly for a language with typologically unusual phonological and phonetic patterns, such as Tashlhiyt, studying how within-speaker and across-context factors affect speech recognition can provide even further insight into how to address the language gap for speech technology.

## Conclusion

In sum, we find that with zero modification, re-using a commercially available Arabic ASR system on Tashlhiyt resulted in close to 45% accurate word transcriptions for clearly spoken voweled words produced by multiple talkers. This means that such an “out-of-the-box” cross-language ASR transfer approach can provide some limited access to spoken language technology for a low resource language. However, the disparities in word transcription (simply in providing a transcription at all, and number of errors when there was a transcription) were systematic: lower performance for vowelless words. Vowelless words are a common word form in Tashlhiyt, so such failures are not trivial. However, future approaches to adapting an Arabic ASR system for Tashlhiyt can be most effective by targeting these linguistic structures. In other words, addressing the language gap in speech technology can be more efficient by understanding the within-language disparities that arise during ASR transfer.

The current study focused on machine recognition for cross-linguistically uncommon word forms—vowelless words in Tashlhiyt—and how differences in phonotactics across target and source language could influence ASR performance disparities. Yet, our findings can be useful for thinking more generally about the factors that affect cross-language ASR transfer. Two languages that have a similar set of consonants and vowels can have quite different word forms and that creates challenges for second language learners: for instance, a Spanish speaker learning English might hear the word ‘sport’ as *esport*, because Spanish does not permit words with initial *s* + obstruent sequences (even though *s* and *p* are common sounds in Spanish)^18,58^. Thus, we predict that in any case where target and source language differ in phonotactics, there will be linguistic disparities. Engineers and scientists can more effectively augment cross-language speech technology transfer by understanding the linguistic issues that might lead to disparities within a target language and addressing them, such as training the system on the types of words that are more likely to fail using this approach. Investigating ways to make ASR more accessible for people who speak low resource languages is one step toward addressing major inequities in access to speech technology across the world. Our study also highlights how linguistically- and phonetically-informed approaches to this aim provide ways to more effectively and efficiently adapt existing speech technology to low-resource languages.

Finally, we find that disparities in ASR transfer generally parallel the types of difficulties that adults make when learning a second language (i.e., difficulties with sound sequences that are illegal in the first language). Therefore, this work can also speak to broader issues in cognitive science, linguistics, and human–computer interaction, particularly in understanding the extent to which machines are similar to or different from humans in mapping the acoustic signal to discrete linguistic representations^29^.

## Ethics and consent

Research was performed in accordance with the UC Davis IRB (IRB number 1407306).

## Data availability 

Data and code available at: https://osf.io/vcqfk/

### Supplementary Information

Supplementary Information.
