# Supplementary material for: Linguistic disparities in cross-language automatic speech recognition transfer from Arabic to Tashlhiyt
Source: Sci Rep. 2024 Jan 3;14:313. doi: 10.1038/s41598-023-50516-3 (PMC10764819; doi:10.1038/s41598-023-50516-3)
Supplement: Supplementary file 1 — Supplementary Information. [file 41598_2023_50516_MOESM1_ESM.docx]

**Supplementary Materials**.

List of items used in the study, with English glosses, and ground truth Arabic transcriptions. The sonority value of the center consonant for vowelless words (following Parker, 2002: 6 = liquids, 5 = nasals, 4 = voiced fricatives, 3 = voiceless fricatives; 2 = voiced stops; 1 = voiceless stops) is provided in parentheticals after the gloss.

| **Voweled Words** | | | **Vowelless words** | | |
| --- | --- | --- | --- | --- | --- |
| **Tashlhiyt word - IPA transcription** | **English gloss** | **“Ground truth” Arabic transcription** | **Tashlhiyt word - IPA transcription (center consonant sonority value)** | **English gloss** | **“Ground truth” Arabic transcription** |
| daʁ | again | داغ | bsr (3) | spread | بسر |
| fan | they gave | فان | žhd (3) | be strong | جهد |
| fat | give 2MS.PL | فات | sdˤr (2) | fall/make drop | سضر |
| fin | they suppurated | فين | bdr (2) | mention | بدر |
| fuħ | revel in | فوح | bzg (4) | swell | بزج |
| lfal | omen | لفال | bʕdˤ (4) | them (emphatic) | بعض |
| lfil | elephant | لفيل | dbʁ (2) | tan | دبغ |
| lždid | new | لجديد | mnʕ (5) | prohibit/forbid | منع |
| lždud | ancestors | لجدود | ʁbr (2) | disappear | غبر |
| man | which | مان | ʁdr (2) | betray | غدر |
| mun | accompany someone | مون | žbd (2) | pull | جبد |
| ʕum | swim | عوم | zdm (2) | collect wood | زدم |
| nuf | we are better | نوف | zbr (2) | prune | زبر |
| ruħ | go home | روح | zgr (2) | cross | زجر |
| ʁar | only | غار | fkt (1) | give it | فكت |
| ʁir | only | غير | fst (3) | feed on | فست |
| sut | drink it | سوت | ftħ (1) | operate | فتح |
| tuf | she's better | توف | ħkm (1) | govern/judge | حكم |
| ʕif | get tired of | عيف | kšf (3) | be faded | كشف |
| ruħ | go home | روح | skr (1) | do/make | سكر |
| sir | go! | سير | ngr (2) | between | نجر |
| zud | like, as | زود | nkr (1) | wake | نكر |
| mit | what | ميت | nšf (3) | scrape | نشف |
| zˤurˤ | visit | زور | nsˤħ (3) | advise | نصح |
| luħ | throw | لوح | nžħ (4) | pass a test | نجح |
| ran | they want | ران | rbħ (2) | win | ربح |
| sul | stay alive | سول | rdˤl (2) | borrow/lend | رضل |
| sak | pass through | ساك | rgl (2) | lock | رجل |
| liʁ | I married | ليغ | rħl (3) | leave the city | رحل |
| tid | these.FM | تيد | frˤħ (6) | be happy | فرح |
| sin | two | سين | ħrm (6) | deprive | حرم |
| lan | they have | لان | krf (6) | tie | كرف |
| ʁir | only | غير | slt (6) | leave on the sly | سلت |
| tut | she hit | توت | tlf (6) | get mixed up | تلف |
| dar | at | دار | žld (6) | leather | جلد |
| gan | they are | جان | zlm (6) | glance | زلم |
| riʁ | I want | ريغ | ʕlf (6) | feed | علف |
